# Mitochondria in the Spotlight: *C. elegans* as a Model Organism to Evaluate Xenobiotic-Induced Dysfunction

**DOI:** 10.3390/cells12172124

**Published:** 2023-08-22

**Authors:** Airton C. Martins, Miriam B. Virgolini, Daiana Silva Ávila, Pablo Scharf, Jung Li, Alexey A. Tinkov, Anatoly V. Skalny, Aaron B. Bowman, João B. T. Rocha, Michael Aschner

**Affiliations:** 1 Department of Molecular Pharmacology, Albert Einstein College of Medicine, Bronx, NY 10461, USA; airton.dacunhamartinsjunior@einsteinmed.edu; 2 Departamento de Farmacología Otto Orsingher, Facultad de Ciencias Químicas, Universidad Nacional de Córdoba, Córdoba 5000, Argentina; 3 Instituto de Farmacología Experimental de Córdoba-Consejo Nacional de Investigaciones Técnicas (IFEC-CONICET), Facultad de Ciencias Químicas, Universidad Nacional de Córdoba, Córdoba 5000, Argentina; 4 Laboratory of Biochemistry and Toxicology in Caenorhabditis Elegans, Universidade Federal do Pampa, Campus Uruguaiana, BR-472 Km 592, Uruguaiana 97500-970, RS, Brazil; 5 Department of Clinical and Toxicological Analyses, Faculty of Pharmaceutical Sciences, University of São Paulo, São Paulo 05508-000, SP, Brazil; 6 College of Osteopathic Medicine, Des Moines University, Des Moines, IA 50312, USA; 7 Laboratory of Ecobiomonitoring and Quality Control, Yaroslavl State University, Yaroslavl 150003, Russia; 8 Laboratory of Molecular Dietetics, IM Sechenov First Moscow State Medical University (Sechenov University), Moscow 119435, Russia; 9 Peoples Friendship University of Russia (RUDN University), Moscow 117198, Russia; 10 School of Health Sciences, Purdue University, West Lafayette, IN 47907-2051, USA; 11 Departamento de Bioquímica e Biologia Molecular, Centro de Ciências Naturais e Exatas, Universidade Federal de Santa Maria, Santa Maria 97105-900, RS, Brazil

**Keywords:** arsenic, *C. elegans*, cadmium, manganese, mitochondria, mercury, ethanol, pesticides

## Abstract

Mitochondria play a crucial role in cellular respiration, ATP production, and the regulation of various cellular processes. Mitochondrial dysfunctions have been directly linked to pathophysiological conditions, making them a significant target of interest in toxicological research. In recent years, there has been a growing need to understand the intricate effects of xenobiotics on human health, necessitating the use of effective scientific research tools. *Caenorhabditis elegans* (*C. elegans*), a nonpathogenic nematode, has emerged as a powerful tool for investigating toxic mechanisms and mitochondrial dysfunction. With remarkable genetic homology to mammals, *C. elegans* has been used in studies to elucidate the impact of contaminants and drugs on mitochondrial function. This review focuses on the effects of several toxic metals and metalloids, drugs of abuse and pesticides on mitochondria, highlighting the utility of *C. elegans* as a model organism to investigate mitochondrial dysfunction induced by xenobiotics. Mitochondrial structure, function, and dynamics are discussed, emphasizing their essential role in cellular viability and the regulation of processes such as autophagy, apoptosis, and calcium homeostasis. Additionally, specific toxins and toxicants, such as arsenic, cadmium, and manganese are examined in the context of their impact on mitochondrial function and the utility of *C. elegans* in elucidating the underlying mechanisms. Furthermore, we demonstrate the utilization of *C. elegans* as an experimental model providing a promising platform for investigating the intricate relationships between xenobiotics and mitochondrial dysfunction. This knowledge could contribute to the development of strategies to mitigate the adverse effects of contaminants and drugs of abuse, ultimately enhancing our understanding of these complex processes and promoting human health.

## 1. Introduction

Mitochondria are double-membrane organelles in eukaryotes frequently referred to as the powerhouses of cells [1]. Cellular respiration relies on mitochondrial complexes that lead to ATP generation through oxidative phosphorylation and the tricarboxylic acid cycle [1,2]. In addition to energy sources, mitochondria play crucial roles in regulating cell signaling, growth, and death [3]. Indeed, mitochondrial dysfunction is directly associated with pathophysiological conditions owing to its pivotal role in coordinating cell fate. Several environmental pollutants and drugs can alter the mitochondrial fine-tuned balance and trigger cellular dysfunction [4,5,6].

The increasing need to understand the intricate effects of contaminants and drugs of abuse on human health requires the use of effective scientific research tools. The nematode *Caenorhabditis elegans* (*C. elegans*) is a powerful model widely used to investigate several phenomena underlying toxic mechanisms since *C. elegans* displays remarkable homology (up to 80%) with the mammalian genome [7,8]. *C. elegans* has been extensively employed to address the mechanism of mitochondrial dysfunction in the pathophysiology of neurodegenerative disorders, and behavioral alterations, and to elucidate the impact of contaminants on mitochondrial functioning [4,9].

Here, we review the effects of several contaminants and drugs of abuse on mitochondria using the experimental model of *C. elegans* and highlight the potential of the nematode to address investigations regarding mitochondrial dysfunction induced by xenobiotics.

## 2. Mitochondrial Dysfunction

### 2.1. General Aspects

The mitochondria is a small organelle composed of two phospholipid bilayer membranes, a highly selective external and an internal with multiple invaginations, tubular or lamellar structures called cristae, which are connected by narrow tubular structures, the cristae junctions [10]; it is filled with a matrix in which several enzymes reside, as well as proteins and mitochondrial DNA that encodes around 13 proteins in humans [11]. It has been posited that this organelle originated from the fusion of phagocyted aerobic bacteria which were not digested by a host anaerobic cell (probably methanogenic archaea) and were then preserved by endosymbiosis [12]. The mitochondria became the cell powerhouse by optimizing ATP production through the coupling of the electron transport chain and oxidative phosphorylation. Notably, this organelle can also control processes such as autophagy, apoptosis and calcium homeostasis [13]. In addition, mitochondria form a dynamic network that changes in shape, size and morphology in response to distinct events, including energetic demands, various stressors, and aging. These dynamics are mediated by fission and fusion, as well as the processes of mitochondrial transport, biogenesis, and degradation [14]. These functions and processes are conserved in *C. elegans*, as are genes related to the Krebs cycle, electron transport chain (ETC), oxidative phosphorylation, lipolysis and apoptosis, just to name a few [14]. Notably, intact and functional mitochondria are essential for eukaryotic cell viability.

However, several conditions can impair mitochondrial function and lead to mitochondrial dysfunction in vertebrates and invertebrates as well. This process is characterized by the loss of organelle efficiency in producing ATP; however, other processes that depend on the mitochondria, such as proteostasis, will be impaired [15].

DNA mutations and damage: Mitochondria have their own DNA (mtDNA), but nuclear DNA also codifies important mitochondrial proteins. These mutations can interfere with the optimal functioning of mitochondrial proteins in ATP production, leading to their dysfunction in *C. elegans* as well [16]. Furthermore, drugs that can cause irreparable mtDNA damage such as mycotoxin aflatoxin B1 and the chemotherapeutic cisplatin, cause increased dopaminergic damage in worms [17]. In this regard, we will explore in the next topic some mitochondrial gene mutations that cause human diseases and can be modeled in *C. elegans*.

Oxidative stress: The presence of oxygen in the mitochondria makes it the cell center for reactive oxygen species (ROS) production. To prevent the amplification of chain ROS production, mitochondria have antioxidant enzymes such as superoxide dismutase [18]. However, some events can trigger an imbalance between prooxidants and antioxidants and trigger mitochondrial dysfunction. Factors such as mutations, exposure to toxicants, aging, immune response and diseases such as cancer and diabetes induce oxidative stress or other processes that lead to lipid peroxidation, protein oxidation, DNA damage, apoptosis activation and opening of the mitochondrial permeability transition pore (mPTP), resulting in loss of mitochondrial membrane integrity and membrane potential [14].

Defects in the electron transport chain (ETC): The ETC is responsible for the generation of the proton gradient that will drive ATP formation in complex V or F1/F0 ATP synthase. Mutations, deficiencies or inhibition of the proteins involved in the ETC, such as Complex I, Complex III, or Complex IV, can impair the electron flow, alter the mitochondrial membrane potential (Δψm) generated by proton pumps and reduce ATP production [19]. Some mutations in genes related to the ETC and oxidative phosphorylation may disturb mitochondrial function and make worms more vulnerable to stress. For instance, *mev-1* mutation (an ortholog of human (succinate dehydrogenase complex subunit C) reduces *C. elegan*’s lifespan and increases oxidative stress [20]. In addition, it has been found that a mutation in a subunit of F-ATP synthase (OSCP) initiates the opening of the mPTP and shortens the worms’ lifespan [21].

Mitochondrial membrane permeability: Changes in the permeability of the inner and outer mitochondrial membranes can disrupt the electrochemical gradient required for ATP synthesis. This can be caused by alterations in the expression or function of proteins involved in maintaining mitochondrial membrane integrity, by oxidative stress, immune response, calcium influx and toxicants, but other factors can also induce permeability alterations [22]. For example, the mPTP is a non-selective channel in the inner mitochondrial membrane permeable to molecules smaller than 1.5 kDa. It includes both mitochondrial F1FO (F)-ATP synthase and adenine nucleotide translocase (ANT), with matrix cyclophilin D (CypD), facilitating the transition to the pore-forming conformation [23]. Its opening is regulated by a variety of factors, such as high levels of calcium and ROS, and the presence of certain drugs or toxins [24,25]. One inhibitor of the mPTP is cyclosporin A, which has been demonstrated to extend *C. elegans* lifespan and increase stress resistance [26]. The alteration in mitochondrial membrane permeability can also be caused by the apoptotic process induced by the intrinsic pathway. Internal cellular signals, such as DNA damage or oxidative stress can initiate the intrinsic pathway, leading to the activation of pro-apoptotic proteins [27]. These pro-apoptotic proteins can promote mitochondrial outer membrane permeabilization, causing the release of cytochrome c and other apoptotic factors from the mitochondria into the cytoplasm. In *C. elegans*, EGL-1, CED-9, CED-4, and CED-3 are the core executioners of worm apoptotic cell death, with CED-9 localized in the outer mitochondrial membrane. Furthermore, the process does not involve cytochrome c release, indicating that the mitochondrial involvement is still little known in worms [28].

Impaired calcium homeostasis: Mitochondria play a vital role in regulating cellular calcium levels by sequestering and buffering cytosolic calcium. The endoplasmic reticulum is responsible for storing calcium and there is a mitochondrial communication that allows its uptake to control the levels [29]. Calcium is important for mitochondrial function, as it activates enzymes from the TCA cycle and increases ATP production [30]. However, disturbances in this process cause massive calcium influx from the outer and inner membranes transporters, overwhelming mitochondrial calcium capacity. Mitochondrial calcium overload, which, when combined with other stressors such as oxidative damage, results in the formation and opening of the mPTP [31,32]. This has been evidenced in *C. elegans* presenilin mutants, which presented neuronal and behavioral defects dependent on elevated mitochondrial calcium-induced oxidative respiration and concomitant ROS generation [33]. Mutations in presenilin genes cause the altered expression of endoplasmic reticulum calcium transporters, thereby increasing cytosolic calcium levels [34].

Mitophagy defects: Mitophagy is a process by which damaged or dysfunctional mitochondria are selectively degraded and removed. In *C. elegans*, mitophagy is essential for development and defects in this process can render worms less resistant to toxins [35] in addition to leading to progressive accumulation of the dysfunctional mitochondrial mass during aging, resulting in persistent cell malfunctions [36]. Drugs such as haloperidol and sevoflurane can impair mitophagy and cause neurotoxicity [37].

Impaired mitochondrial dynamics: The mitochondrial dynamics rely on biogenesis (mitochondrial division by mtDNA replication); fission (the division of the mitochondria, without mtDNA replication) and fusion (the association of two mitochondria for content exchange). These processes maintain mitochondrial homeostasis, regulate mitochondrial form, volume and function, and are increasingly understood to be critical components of the cellular stress response. Several studies have demonstrated that deficiencies in the genes related to these processes render worms less resistant to toxicants such as paraquat, acrolein, rotenone and cisplatin [38].

Aging: The ETC reactions throughout the whole lifetime of any species are a source of continuous electron leakage, and incomplete oxygen reduction at Complex IV during *C. elegans* life leads to ROS production and oxidative stress, as occurs in mammals [14]. As evidence, supplementation with antioxidants can extend the nematode’s longevity by attenuating oxidative stress and aging-related endpoints such as locomotion and lipofuscin levels [39]. Furthermore, the accumulation of mutations in mitochondrial DNA [40] and the deregulation of the mitochondrial unfolded protein response (UPRmt) [41] contribute to the aging process in worms.

Immune response: The mitochondria play an important role in the innate immune response as they are targets for virulence factors and microbial toxins seeking hemes and iron–sulfur proteins, triggering a mitochondrial stress response that may counteract the infection [42]. Indeed, excessive or chronic inflammatory responses induce ROS production in the mitochondria to act as signaling molecules and to activate immune cells [43]. For instance, the pathogenic bacteria *Pseudomonas aeruginosa* secrete multiple toxins capable of perturbing oxidative phosphorylation (OXPHOS), including cyanide, siderophores, and phenazines, which impair OXPHOS Complex IV, host iron acquisition, and electron transport, respectively [44,45]. Furthermore, cytokines such as tumor necrosis factor (TNF)-alpha and Interleukin-1 (IL-1), can activate the nuclear factor-kappa B (NF-κB) pathway and alter the mitochondrial biogenesis and dynamics as well [46]. Notably, *C. elegans* lives naturally in a microbe-rich environment [47]. The mitochondrial unfolded protein response (mUPR) is induced to protect against pathogens [48]; however, if this system fails, there is repression of this response and increased ROS production, thereby leading to the nematode’s death [49]. In addition, pathogens such as *Bacillus thuringiensis* have a toxin that opens pores in the mitochondria, resulting in the loss of mitochondrial potential and its dysfunction [49]. In contrast, it has been demonstrated that mild mitochondrial dysfunction induced by Complex I inhibitor rotenone can activate the p38 MAPK-mediated immune pathway in intestinal cells, which was protective to dopaminergic neurons [50].

In this regard, several environmental toxins, drugs, or chemicals can directly affect mitochondrial function. For example, exposure to heavy metals, pesticides, or medications may impair mitochondrial activity (inhibit ETC, induce mPTP, alter the expression of chaperones and antioxidant genes, and/or induce mtDNA mutations [51] For instance, airborne exposure to the solvent toluene in *C. elegans* triggered dopaminergic and cholinergic damage, associated with reduced Complex I activity, lower ATP synthesis, apoptosis in the germline cells and loss of mitochondrial mass [52,53]. The docking analysis further indicated that toluene could interact with the PSST look of NADH dehydrogenase (Complex I), reducing its activity. The effects of metals, metalloids and organic molecules will be further explored in this review.

### 2.2. Human-Related Mitochondrial Disorders Modeled in C. elegans

Mitochondrial diseases are a class of severe conditions. Because mitochondria is essential to ATP production in most of the tissues, especially in the central nervous system and muscles, the symptoms are mostly related to the functions they control: loss of muscle coordination, muscle weakness, neurological deficits, including seizures, learning disabilities, visual and hearing problems [12]. Moreover, developmental delay, respiratory issues, thyroid and adrenal dysfunction may also be observed. In addition to mitochondrial diseases caused by mutations in the genes required for mitochondrial function, mitochondrial defects have been linked to a variety of age-related disorders, including neurodegenerative diseases, cardiovascular diseases, cancer, and diabetes [54].

As previously mentioned, the mitochondrial machinery of *C. elegans1* is highly similar to that of mammals, as are the processes this organelle controls, and the mechanisms by which its dysfunction occurs. Because the *C. elegans* genome is completely known and the generation of mutants is easy, especially with the advance of CRISPR techniques, modeling mitochondrial diseases in this nematode has become a great tool for drug discovery or to study the interaction with toxicants. These mutants have been recently reviewed to a great extent by Onraet and Zuryn (2023) [14]. Therefore, here, we will focus on mitochondrial dysfunction models and their relationship with metals, metalloids and organic molecules.

A Complex II deficiency has been associated with a mitochondrial disease called Leigh syndrome which results in metabolic changes leading to lactic acidosis [55]. The Complex II enzyme succinate dehydrogenase catalyzes the electron transport from succinate to ubiquinone and is composed of four subunits, one of them named Cyt-1/ceSDHC, which consists of a cytochrome b560 that binds to ubiquinone. Mutations in this subunit have been related to the inherited propensity for developing head and neck tumors [56]. In *C. elegans*, the homologous subunit is MEV-1 and mutations in *mev-1* cause increased ROS production, increased lipofuscin, protein carbonylation [57] and induction of the apoptotic pathway, thereby reducing the worms’ lifespan [58]. These mutants are hypersensitive to triclosan, a powerful antimicrobial chemical with potential endocrine-disrupting properties, as demonstrated by increased ROS levels and reduced lifespan [59]. Furthermore, *mev-1* mutants presented reduced ATP levels following exposure to arsenite, which was more significant in relation to other ETC mutants (*nuo-6* for Complex I, *isp-1* for Complex III, and *atp-2* for Complex V) [60].

CISD/NEET are mitochondrial iron–sulfur cluster binding proteins, and mutations in their genes have been implicated in the pathogenesis of Wolfram neurodegenerative syndrome type 2, a rare and devastating disorder [61]. *cisd-1* is the single ortholog of CISD1/MitoNEET and CISD2/NAF-1 in *C. elegans*, and mutants display common features observed in mammals such as damage to motor neurons, disruption of ETC, reduced lifespan and altered proteostasis, as evidenced by increased neurodegeneration in aggregation-prone nematodes expressing polyQ40 (Huntington’s disease) and mutated alfa-synuclein (Parkinson’s disease) [15]. Exposure to the pesticide paraquat, a superoxide radical anion generator which inhibits Complex I, promoted increased ROS production in relation to wildtype worms, which was mitigated by overexpression of CISD-1 [62]. Of note, overexpression of CISD-1 also promoted higher phosphorylation of PMK-1 following paraquat exposure, indicating a promotion of the immune response against pathogens [62].

Mutations in genes related to mitochondrial dynamics such as fission (DRP1), fusion (MFN2, OPA1, FZO-1), and mitophagy (PINK1, PARK2) are associated with cardiac, neuronal diseases, and cancer, just to name a few [63,64]. The major executor of fission is the dynamin-related protein 1 (DRP1). DRP1 is mainly cytosolic but translocates to the mitochondrial surface in order to mediate fission of the organelle. In *C. elegans*, overexpression of *drp-1* causes mitochondrial fragmentation [65], whereas the knockout of this gene causes the mitochondrial matrix to retract into large blebs that are both surrounded and connected by tubules of outer membrane [65]. These mutants showed higher sensitivity to several toxicants, particularly, reduced survival following paraquat, arsenite and rotenone exposure, and reproductive toxicity caused by paraquat and arsenite [66].

Mitochondrial fusion is a process that requires outer and inner membrane proteins to merge two mitochondria, which will exchange material and promote tubular and elongated mitochondria, improving its function [38]. This process is so important that mitofusin (MFN2) mutation causes Charcot-Marie-Tooth neuropathy type 2A, while optic atrophy 1 (OPA1) mutations cause dominant optic atrophy [38]. In *C. elegans*, the homologous proteins are FZO-1 and EAT-3, respectively. Mutations in these genes caused delayed larval growth following exposure to aflatoxin B1, cisplatin and at a higher extent, to arsenite, which also caused reduced oxygen consumption and ATP levels in worms at the L4 stage [66].

Damaged mitochondria can be eliminated via mitophagy, as the loss of membrane potential results in the accumulation of PTEN-induced putative kinase 1 (PINK1) on the outer membrane. PINK1 phosphorylates outer mitochondrial membrane proteins, including MFN2, and recruits the E3 ubiquitin ligase parkin (PARK2) from the cytosol. Parkin then ubiquitinates these proteins targeting the mitochondrion for autophagic degradation [67]. Indeed, mutations in PINK1 and PARK2 are associated with early-onset familial Parkinson’s disease (PD), PARK2 mutations being responsible for about 40% of the cases [68]. These features have been replicated in *C. elegans* mutants lacking PD-related genes. Loss of the mitophagy genes *pink-1* and *pdr-1/parkin* results in heightened susceptibility to neurodegeneration caused by 6-hydroxy- dopamine (6-OHDA), a chemical model of dopaminergic and noradrenergic neurodegeneration in Parkinson’s disease [69]. Furthermore, *pdr-1* mutants accumulate more manganese than wildtype worms and present higher ROS levels [70]. These mutants presented no alteration in the expression of divalent metal transporters, but a decrease in the efflux transporter ferroportin [71], resulting in increased Mn levels. High Mn levels are threatening as this metal accumulates in dopaminergic neurons and causes neuronal damage in humans and in *C. elegans* as well [7].

Altogether, these data indicate that environmental or occupational exposure to toxicants can, in addition to inducing mitochondrial dysfunction (which will be further explored in the next sections), also accelerate the progression of genetic mitochondrial diseases or complicate the symptoms by aggravating mitochondrial damage.

## 3. *C. elegans* and Specific Toxins/Toxicants

### 3.1. Metals/Metalloid

The presence of harmful chemicals, especially hazardous metals in the environment, remains an important public health concern [72,73]. In this section, we describe how mitochondrial dysfunction can be impacted by metals/metalloids and the importance of *C. elegans* strains in deciphering the mechanism involved. Moreover, we also describe the involvement of drugs of abuse and pesticides in mitochondrial dysfunction.

#### 3.1.1. Arsenic

Arsenic (As) is a metalloid that is widely present in the environment due to geologic and anthropogenic sources. This element is recognized as an important toxicant, with As ranked as the first substance in the Hazardous Substance Priority List established by the US ATSDR [74]. Moreover, it is recognized as a carcinogenic and genotoxic element [75,76]. The major route of exposure to As are contaminated water and food such as rice and other cereals [77,78]. Other sources of exposure are industrial mining and refining activities, such as the smelting of metals, production of pharmaceuticals, manufacturing of pesticides [79,80].

Oxidative stress is a major result of the induced toxic effects after As exposure. Several studies reported that As induced an increase in ROS, such as superoxide anions, hydroxyl radicals, hydrogen peroxide, singlet oxygen and peroxyl radicals and As also induced the depletion of antioxidant defense enzymes, such as SOD, CAT, GPx, as well as the depletion of GSH [81,82,83,84]. Mitochondria are the primary target of As-induced toxic effects, indirectly via ROS accumulation or directly via the condensation of the mitochondrial matrix and the opening of permeability transition pores [85].

*C. elegans* has been instrumental in addressing As-induced toxicity. Low-dose arsenite was shown to increase ROS formation, promoting the upregulation of mitochondria proteins to increase the lifespan, while higher concentrations reduced longevity, demonstrating that As can modulate the lifespan through the activation of mitochondrial ROS formation by a process named mitochondrial hormesis [86].

Using the *C. elegans* model, Luz et al. (2016) reported that the metalloid induced an alteration in mitochondrial function, including the reduction in ATP-like respiration and spare respiratory capacity and augmented proton leaks, whereby worms deficient in electron transport chain Complexes I, II, and III, but not ATP synthase, were sensitive to As exposure [87]. The same group tested whether exercise afforded protection against mitochondrial dysfunction and lethality, demonstrating that after a 24 h exposure of arsenite, an exercised animal had improved mitochondrial health and lower lethality. In agreement with this, exercise protected worms from rotenone, which is a strong inhibitor of mitochondrial respiratory Complex I, and extended the worms’ lifespan. Further, the authors suggested that exercise conditioning could protect mitochondrial dysfunction against As exposure and age-related decline in mitochondrial health [88].

Important processes such as mitochondria fission, fusion and mitophagy are related to the stress response in response to As exposure [89]. Nematode exposure to low-dose As, which acts as a mithormetic substance, led to improved mitochondrial function. Moreover, with *C. elegans* mutant strains deficient in mitochondria fusion and fission, it was shown that those strains were more sensitive to As than the wild-type [66]. Additionally, As exposure preferentially disrupted the mitochondrial function in fusion-deficient worms, suggesting that the disruption of the pyruvate metabolism and Krebs-cycle activity trigger the mitochondrial deficits [66].

#### 3.1.2. Cadmium

Cadmium (Cd) is a toxic and non-essential heavy metal that is recognized as a threat to human health. Cd exposure can occur through contaminated food and water intake, occupational inhalation, or cigarette smoking [90,91]. Upon absorption, Cd accumulates in target organs (including the liver and kidneys) and has a long half-life of up to 10 years, which contributes to its high toxicity [92]. Chronic Cd exposure is associated with the emergence of a myriad of diseases, including hypertension, renal failure, neurodegenerative disorders, and cancer [93,94].

Impaired autophagy, DNA damage, and redox signaling imbalances are well-recognized mechanisms of Cd toxicity [92,95]. Notably, the latter phenomenon has been extensively investigated. Although oxidative stress is a hallmark of Cd poisoning, Cd is not a Fenton-like metal; hence, Cd is not a direct ROS generator but modulates other pro-oxidative sources that result in the overproduction of superoxide and hydrogen peroxide [96,97]. As Cd leads to mitochondrial dysfunction, Cd-associated ROS generation has been attributed to mitochondrial impairments [98].

Indeed, mitochondria are direct targets of Cd-induced toxicity. Once in the mitochondria, Cd induces ROS production by inhibiting Complex III, the mitochondrial complex most sensitive to Cd [98]. Beyond ROS generation per se, Cd also depletes the enzymatic (catalase, GPx, and SOD) and non-enzymatic (GSH and metallothioneins) antioxidant defense machinery [99,100]. Consequently, an insufficient anti-oxidative apparatus and increased ROS generation trigger oxidative stress and altered redox state, and mediate intracellular stress responses and macromolecular damage. Cd blocks the ETC by impairing electron flow through the complexes, disrupting mitochondrial respiration by increasing the inner membrane permeability and decreasing the mitochondrial membrane potential [98,100,101]. Due to the Cd-induced blockage of ETC, there is excessive mitochondrial ROS production, which can cause the opening of the permeability transition pore, DNA damage, and mutations [102,103].

Based on the potential genotoxic effect caused by Cd and the poor DNA repair machinery in mtDNA, Leuthner et al. employed *C. elegans* to assess the potential of cadmium chloride (CdCl_2_) to induce mtDNA damage and mutagenesis [104]. Interestingly, CdCl_2_ led to higher levels of damage in mtDNA than in nuclear DNA, which was also reported by González-Hunt and collaborators [17]; however, chronic CdCl_2_ exposure failed to induce single-nucleotide mutations in wild-type worms [104]. Since mitophagy is a crucial process in removing damaged mitochondria, the authors tested the impact of CdCl_2_ in the mitophagy-deficient strains *pink-1* and *dcl-1*. Only the *pink-1* strain showed higher levels of mtDNA damage induced by Cd; however, no mutations were detected [104]. Associated data demonstrated that CdCl_2_ causes mtDNA damage accumulation, but *C. elegans* is resistant to CdCl_2_-induced mtDNA mutagenesis via a mitophagy-independent mechanism, which highlights other potential evaluative mechanisms of mtDNA repair [104].

Cd can disrupt mitochondrial morphology, leading to mitochondrial fragmentation, non-functional mitophagy, and the inhibition of ATP synthase [98,100]. An imbalance in the mitochondrial respiratory chain function is a hallmark of impaired cellular metabolism associated with neurodegenerative diseases and cancer onset and progression [105,106]. To evaluate how environmental toxicants could alter the oxygen consumption rate (OCR), worms exposed to Cd showed reduced OCR in a dose-dependent manner [107,108]. Interestingly, OCR suppression due to Cd exposure is positively associated with worm growth inhibition [108].

**Figure 1:**
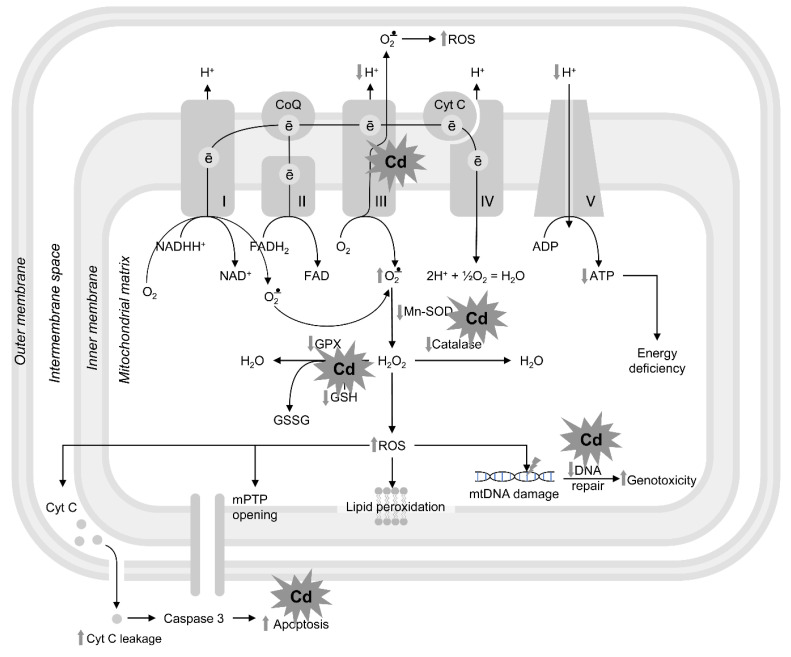
The mechanisms of Cd-induced mitochondrial dysfunction. Cadmium affects Complex III of the mitochondrial respiratory chain resulting in electron leakage and subsequent superoxide formation. Complex I may also contribute to the production of superoxide in mitochondrial matrix. Cd-induced disturbances in electron transport chain also results in reduced proton gradient and inhibition of ATP-synthase (Complex V), ultimately leading to reduced ATP production and energy deficiency. In addition to increased superoxide production, Cd exposure also reduces mitochondrial antioxidant system by depleting non-enzymatic antioxidant pool (GSH, thioredoxin) and inhibiting mitochondrial antioxidant enzymes. Excessive mitoROS generation upon Cd exposure induces lipid peroxidation, promotes mitochondrial permeability transition pore opening, as well as increases in mitochondrial cytochrome c leakage and subsequent apoptosis activation through mitochondrial pathway. Cd-induced ROS overproduction also results in DNA damage that is further aggravated by inhibition of DNA reparation mechanisms, contributing to mtDNA mutagenesis.

Notably, using transcriptional profiling and data-modeling approaches, Swain et al. reported that *C. elegans* exposed to Cd displayed an altered physiological mode of action and transcriptional signature, with alterations particularly focused on stress response and energy metabolism [109]. The authors reported that Cd exposure induced transcriptional alterations in several genes related to ATP turnover and mitochondrial biogenesis and functioning [109], which reinforces mitochondria as a direct target of Cd. The mechanism of Cd-induced mitochondrial dysfunctions is illustrated in Figure 1.

#### 3.1.3. Manganese

Manganese (Mn) is a ubiquitous trace metal that can be found in our environment and commonly acquired through our diet. In the appropriate amount, this metal is involved in many key biological processes such as the synthesis and activation of enzymes (e.g., oxidoreductase, transferase), metabolism of glucose and lipids, as well as providing protection from oxidative stress [110]. However, while some Mn is essential for our body to maintain homeostasis, excessive amounts of Mn have been linked with many neurodegenerative diseases such as Parkinson’s disease (PD), Alzheimer’s disease (AD), and Huntington’s disease (HD), as well as several metabolic syndromes [111].

While the relationship between Mn and these disease processes is poorly understood, there is ongoing research to suggest that its accumulation and disruption of mitochondrial function may play a crucial role in the pathogenesis of several disease states [112]. It is well established that under aerobic conditions, mitochondria generate superoxide radicals via the consumption of oxygen. Mitochondria have several antioxidant defense systems (e.g., superoxide dismutase, glutathione peroxidase) that allow it to tolerate the excess ROS. However, when these defense systems are exhausted, a state of oxidative stress is created, causing mitochondrial damage.

Brown et al. proposed several mechanisms by which Mn can disrupt mitochondrial function: by inhibition of energy transduction, increased generation of free radicals, or induction of mutations in the mitochondrial genome [113,114]. This proposal has also been supported by several in vivo studies such as that by Galvani et al., which demonstrated Mn’s inhibition of the mitochondrial electron transfer chain in several neuronal cell lines [115]. This disruption of the electron transport chain may enhance the generation of free radicals and induce mutations in the mitochondrial genome as well. Furthermore, there is also evidence to suggest that excessive Mn also disrupts mitochondrial [Ca^2+^] leading to calcium overload and cellular damage under pathological conditions [114].

*C. elegans* has been used as a model in exploring the pathogenesis of many neurodegenerative diseases. Cooper et al. demonstrated in their study using *C. elegans* that there exists a relationship between the dysfunctions of mitochondrial genes (e.g., PINK1) and the pathogenesis of PD [116]. However, despite evidence of Mn’s deleterious effect on mitochondria, future studies using *C. elegans* as a model may provide more insight into the pathogenesis of these disease states.

#### 3.1.4. Mercury

Mercury (Hg) is a heavy metal known for its toxicity. Unlike Mn, however, there exists no known physiological role for Hg in humans. Despite its limited role in our biological processes, common exposure to this heavy metal from the ingestion of contaminated fish and inhalation from occupational exposure can cause a plethora of detrimental effects [117,118]. Several forms of mercury exist, each with its own associated toxicity. Inhaled mercury vapor primarily affects the brain. Mercurous material damages the gut and kidneys, and methyl mercury is widely distributed throughout the body [117].

Exposure to Hg has been demonstrated through rat cell line PC21 to cause impairment of cell viability due to ROS formation and deregulation of intracellular antioxidants associated with mitochondrial dysfunction [119]. Furthermore, Hg exposure induces a dose-dependent mitochondrial swelling and the release of cytochrome c, a hallmark of mitochondrial dysfunction and apoptosis [119]. Even small amounts of mercury have been demonstrated to cause nerve cell changes that are typical for AD [120]. There is also an association between exposure to Hg and the development of ALS, although the connection and pathophysiology is poorly understood [120].

Several studies have characterized the effects of Hg on the developing nervous system using *C. elegans* as a model. Xing et al. demonstrated the degeneration of GABAergic neurons, observed in neurodegenerative diseases such as Huntington’s, after treatment with HgCl_2_ in younger larvae (L1–L3) [121]. Martinez et al. also demonstrated the loss of dopaminergic neurons, observed in Parkinson’s later in life, following early-life (L1) MeHg exposure [122]. Numerous neurobehavioral studies have confirmed MeHg-induced neurodevelopmental toxicity in young C. elegans as well [123]. However, although the relationship between Hg exposure and neurodegenerative changes has been observed, the mechanism of toxicity is still not well understood. The pathogenesis of certain disease states such as AD and HD related to Hg exposure is also not yet been well characterized.

#### 3.1.5. Iron and Cupper

Iron (Fe) is a vital trace element necessary for numerous physiological processes. Its significance extends to critical cellular functions such as mitochondrial support, facilitating the processing of neurotransmitters, enabling effective oxygen transportation, being a fundamental factor in cellular growth, differentiation, DNA synthesis, and the generation of ATP. However, Fe has the capacity to generate reactive oxygen species (ROS) through processes such as the Fenton and Haber–Weiss reactions. These reactions yield a highly reactive hydroxyl radical ion (OH·), which in turn leads to damage in neuronal cells [124]. Furthermore, disruptions in the balance of Fe within the mitochondria result in diminished ATP production, the release of cytochrome c, and the fracturing of mitochondrial structures, thereby causing changes in morphology [125,126]. In this regard, our group recently addressed the involvement of Fe in the *C. elegans* model to investigate molecular mechanisms such as oxidative stress, mitochondrial dysfunction, disrupted homeostasis, and how these factors contribute to neurodegenerative diseases triggered by Fe [127,128].

Indeed, the evaluation of mitochondrial Fe metabolism and mitochondrial ROS demonstrated that suppressing mitoferrin-1 led to a decreased mitochondrial Fe content and a reduction in mitochondrial ROS levels in the CL2006 and GMC101 strains, suggesting that diminishing mitoferrin-1 expression may attenuate features inherent in the advancement of Alzheimer’s disease in *C. elegans*. Moreover, the authors proposed a significant role of mitoferrin-1 in the mitochondrial-Fe metabolism during disease progression [126].

Recently, studies have demonstrated the important role of mitochondrial homeostasis during aging processes. For instance, CISD-1, a mitochondrial iron–sulfur cluster binding protein plays a role in extending the lifespan in *C. elegans*. This extension is achieved via the activation of autophagy and the mitochondrial intrinsic apoptotic pathways. The protein, CED-9, known for its anti-cell-death functions, acts downstream to convey the effects of CISD-1 on maintaining proper protein balance, neuronal health, and overall lifespan. Additionally, maintaining the intracellular levels of Fe in cells is crucial for the proper functioning of CISD-1. Interestingly, even slight increases in Fe supply are able to attenuate the aging process and partially improve impaired mitochondrial energy production and protein balance in *C. elegans* lacking CISD-1 [15]. In addition, Schiavi et al., showed that mitochondria hormesis delays aging by limiting Fe availability in *C. elegans*. The authors suggest that part of this mechanism involves the mitigation of ferroptosis, a type of non-apoptotic cell death triggered by iron-induced lipid peroxidation. This mitigation occurs through interactions with various essential components of ferroptosis and is likely facilitated by the independence of the enzyme GPx redox system [129].

Taken together, the crosstalk between Fe homeostasis and mitochondrial function using the *C. elegans* model has been employed to explore emerging molecular mechanisms, such as ferroptosis, a process implicated in the onset of neurodegenerative disease. Furthermore, the *C. elegans* model offers a platform to investigate mechanisms involving the regulators of Fe transport and the proteins associated with neurodegenerative disorders as well as strategies to promote health with future extrapolation to humans.

Similar to Fe, copper (Cu) is essential for several cell functions, including oxygen metabolism, iron uptake, and ROS detoxification [130]. Cu serves as a cofactor for cytosolic and mitochondrial SOD1, countering ETC-generated ROS, while also aiding in mitochondrial Fe uptake as a cofactor of ferroxidases, vital for FeS cluster assembly and heme biosynthesis [131]. In addition, the assembly of cytochrome c oxidase requires Cu from metallochaperones [131]. Cu overload can occur due to the dysregulation of its metabolism or excessive environmental exposure and lead to cell death [132]. Secondary to the Fenton reaction, Cu is involved in ROS production through different pathways. In this context, Cu ions can trigger mitochondrial dysfunctions by altering the activity of Complex I, II, and IV [133] or by depleting the cellular GHS pool [134], leading to oxidative stress.

In *C. elegans*, Cu-induced toxicity is marked by oxidative stress [135], DNA damage [136], and neurodegeneration [137]. Although there is a lack of studies on Cu-induced mitochondrial dysfunctions in *C. elegans*, several outcomes related to Cu toxicity highlight the role of mitochondria as a potential targets. Transcriptional analysis shows that Cu-exposed worms display an aging-related phenotype marked by differential gene expression in longevity pathways, including mitochondrial respiration and stress [138].

Notably, Cu tolerance was observed in mutants of the divalent metal transporters, smf-1 or smf-2, indicating their involvement in Cu-induced DAergic neurodegeneration, as reported by Mashock et al., in 2016 [137]. The transporter family, smf, assumes a significant role in responding to manganese-induced toxicity [7]. It is plausible that interference with the optimal function of this transporter family imparts the effects of Cu-induced toxicity. The versatility of *C. elegans* as a model organism emerges as a potential tool to untangle the mechanisms by which Cu affects mitochondrial dynamics and exerts its toxicity. Further investigations are required to evaluate the impact of both Cu and Cu-containing mixtures on mitochondria in *C. elegans*.

### 3.2. Drugs of Abuse

Addiction is, phylogenetically, a very ancient process, as many mechanisms underlying addictive behaviors are present in invertebrates [139,140]. This chronic and relapsing brain disorder behaviorally characterized by compulsive seeking despite adverse consequences, involves both heritable and epigenetic mechanisms. Thus, although the prospect of modeling some complex behavioral states, such as “motivation” in *C. elegans*, is debatable [141], there are reports that this organism develops a conditioned preference for cues that had been previously paired with several drugs, including cocaine, methamphetamine, and nicotine [142,143,144]. However, there is little evidence of the adverse effects of drug abuse in *C. elegans*, with significant evidence coming from ethanol (EtOH), as will be detailed below [145,146].

Several studies have been performed in *C. elegans* with psychostimulants. Regarding methamphetamine, it was shown that environmentally relevant levels produce transgenerational cumulative damage in *C. elegans*, evidenced by impaired viability and decreased fecundity [147]. Cocaine, on the other hand, stimulates egg-laying, a behavior that is dependent on acetylcholine functionality [148]. Finally, the amphetamine-induced dopamine efflux through the dopamine transporter (DAT-1) was accompanied by changes in swimming behavior in *C. elegans* [149,150]. Interestingly, a tolerance to the psychostimulant effects was reported after repeated treatment with amphetamine with a more robust impact observed after longer intervals between treatments [151].

Concerning EtOH, *C. elegans* shows adaptive changes evidenced as an enhanced attraction to the drug in a concentration-dependent fashion [152], with increased preference following chronic exposure [146]. Moreover, worms self-exposed to EtOH in a chemoattraction paradigm, even in the absence of prior conditioning [144,153], found their behavior prevented by naltrexone (a pan-opioid antagonist) or varenicline (a nicotinic cholinergic receptor partial agonist), both drugs used in the treatment of alcohol use disorders (AUDs). Aversion-resistant seeking, characterized by a disruption in the control of EtOH intake due to an imbalance between the craving for the drug and the mediation of aversive stimuli, was also recently described in *C. elegans* [154,155]. Notably, following pre-exposure to EtOH, worms showed behaviors characteristic of addiction in humans [156], such as sensitization, tolerance, and withdrawal that could be partially or fully reversed by re-exposure to a low dose of the drug [146,157,158,159]. Based on these antecedents, it is clear that *C. elegans* is an excellent model for identifying both behaviors and molecular mechanisms mediating drug effects as well as potential therapeutical targets [140]. In effect, acute EtOH exposure induces biphasic responses in the living organisms that are considered a sensitive indicator of toxicity [160], and occur at the same internal EtOH concentration that produces intoxication in humans and other mammals [161]. In search for the molecular bases of these behaviors, several reports demonstrate that EtOH activates BK channels encoded by *slo-1*, while mutations in this gene produce resistance to the drug’s effects on locomotion [153,162]. Interestingly, acute functional tolerance is a neuronal plasticity phenomenon in worms, aimed at adapting to the environment [152,163], which is also associated with BK activation [153].

In addition, experimental evidence in *C. elegans* revealed that long EtOH exposure (8 h) had profound effects on the transcriptome, including genes involved in neuronal function, lipid microenvironment, and physiological responses to EtOH. In contrast, short EtOH exposures (up to 2 h) induced the expression of the metabolic enzymes involved in EtOH’s metabolism, in particular, alcohol dehydrogenase, ADH [164], the *sodh-1* reporter gene with equivalent functions to the ADH enzyme [161,165]. Accordingly, the first step in EtOH metabolization occurring in *C. elegans* is slow, reversible, and sustained, with tissular EtOH concentrations not substantially decreasing during a single steady exposure [161,166]. On the other hand, several *alh* genes encode for ALDH, suggesting the mitochondrial isoenzyme is one of the principal actors in EtOH neurotoxicity. In this regard, the gene *gas-1* encodes for nicotinamide adenine dinucleotide (NADH), the ALDH cofactor reoxidized in mitochondrial Complex I, which is crucial for EtOH sensitivity in the nematode [167]. A mutation in *gas-1* causes hypersensitivity to the sedative effects of all straight-chain alcohols up to C12 and volatile anesthetics [168]. Other evidence of EtOH effects in mitochondria was provided by Oh et al., who recently demonstrated fragmentation of this organelle, probably because of fission from the internal membrane [169]. In this line, *dauer* larvae exposed to EtOH survive much longer because EtOH prevents or delays mitochondrial fragmentation and deterioration during energy depletion [170]. Importantly, the amount and time of EtOH exposure in mammals were associated with the severity of mitochondrial dysfunction, starting with an imbalance in the redox response followed by decreased ATP production and the opening of the mitochondrial permeability transition pore (mPTP). Next, decreased activity of the ETC and the loss of mitochondrial membrane potential is observed (hangover), followed by high Ca^2+^ concentrations (chronic toxicity) that finally lead to the prolonged opening of mPTP, triggering neuronal death during withdrawal. It is unknown at present whether these processes can be recapitulated in *C. elegans*, a topic that deserves further study to unravel the functional and structural changes that occur in the mitochondria in response to prolonged EtOH exposure.

In summary, even though *C. elegans* does not allow modeling of the whole spectrum of complexities present in AUDs in humans, this nematode has demonstrated its ability to reproduce important aspects of EtOH toxicity. Furthermore, invertebrates offer many possibilities for advancing the understanding of the behavior, genes, and mechanisms underlying EtOH-induced behaviors. In the first place, the drug is metabolized by mechanisms similar to those in vertebrates and invertebrates show similar signs of intoxication; secondly, the molecular pathways mediating the EtOH actions and/or AUD-related behaviors are similar to those in vertebrates, and third, the behaviors that are used as criteria for determining AUDs (such as tolerance, preference, and reinstatement of EtOH consumption after periods of abstinence), can also be recapitulated in invertebrates [170]. Furthermore, elegant genetic tools available in these models can be used to dissect the causalities between different behavioral components and their contributions to the development and maintenance of AUDs.

### 3.3. Pesticides

These are a diverse class of chemical compounds aimed at controlling pests that are present in low concentrations in the environment with a wide range of properties that determine different modes of toxicity. The nematode *C. elegans* is considered a suitable model to evaluate their adverse effects, with the most studied endpoints being survival, reproduction, redox status, and energy metabolism [171,172,173,174].

The mitochondria, as the powerhouse of the cell, possess a high vulnerability to environmental toxicants [51], with many pesticides affecting the mtDNA or genes associated with changes in bioenergetics. Pesticides are responsible for excessive ROS production, alterations in mitochondrial membrane permeability, calcium homeostasis, ATP production, mitochondrial complex activity, and/or oxygen consumption. Consequently, dysfunctional mitochondria will eventually lead to cell death by apoptosis either by intrinsic pathways involving the mitochondria and DNA damage or through the extrinsic pathway [175].

Thus, there follows a brief description of several pesticides or families of pesticides, with a focus on those reported to affect the cellular redox balance and/or the mitochondrial functionality, providing the tight relationship between these two scenarios that ultimately determine the cell integrity. Additionally, references to dopaminergic neurotoxicity are also included, given the potential association between certain pesticides, mitochondrial dysfunction, and the pathogenesis of Parkinson’s disease (PD) [176].

#### 3.3.1. Paraquat

Paraquat or 1, 1′-dimethyl-4, 4′-bipyridinium ion was developed in the early 1960s as a non-selective contact herbicide that has redox properties [177]. It is poorly absorbed by inhalation, but when ingested orally, causes an acute intoxication episode with severe sickness and death, while chronic exposure has been associated with the etiology of PD [178,179]. Due to its toxicity, paraquat is banned in many countries, but licensed persons are allowed to use it with some restrictions [180]. The main toxic mechanism is related to the interference in the redox cycle of glutathione and thioredoxin generating intracellular ROS, including O_2_^•−^, H_2_O_2_, and HO^•^ [181]. It can also interact with nicotinamide adenine dinucleotide phosphate (NADPH) oxidase (NO_x_) and inducible nitric oxide synthase (iNOS), generating ROS and reactive nitrogen species (RNS) in the cytosol [182]. High levels of NO can react with O_2_^•−^ to form highly toxic peroxynitrite anions (ONOO^–^). Paraquat can also disrupt the oxidation of NAD(P)H to NAD(P)^+^ in the ETC Complex I, by accepting electrons to form paraquat^+^, which in turn can generate O_2_^•−^ and lead to other ROS products such as HO^•^ [183]. Additionally, brain mitochondria ETC Complex III was also shown to be affected by paraquat-induced H_2_O_2_ levels, reducing the mitochondrial transmembrane potential and cytochrome c release, leading overall to mitochondrial dysfunction [184]. Interestingly, a mitochondria-targeted compound, MitoParaquat was developed to be used to selectively increase O_2_^•−^ production within the mitochondrial matrix in vitro and in vivo [185]. Importantly, as *C. elegans* presents a glutathione cycle similar to that of mammals [186], the synthetic antioxidant, N-acetyl cysteine has the ability to prevent paraquat-induced mortality [187], and increase life expectancy in wild-type or Complex I-deficient strains [188], as well as resistance to environmental stressors such as paraquat [189,190]. Moreover, paraquat triggered structural damage in mitochondria, ATP depletion, and increased autophagy in a concentration-dependent manner in transgenic worms with GFP-tagged mitochondria and decreased the expression of genes involved in mitochondrial Complexes I, II, and III in transgenic worms with GFP-tagged mitochondrial Complexes [191].

**Figure 2:**
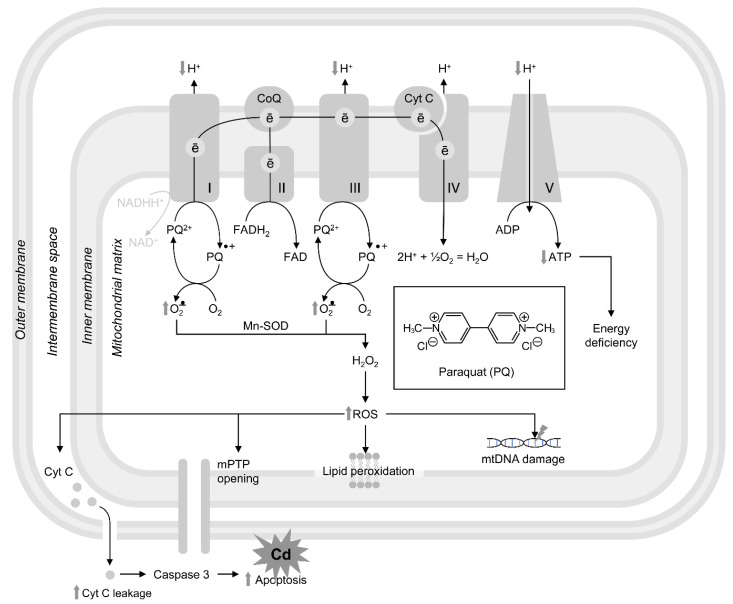
The proposed role of paraquat in mitochondrial dysfunction. Paraquat exposure affects functioning of mitochondrial electron transport chain through its interaction with Complex I by accepting electrons with the formation of paraquat radical (PQ^+•^). The latter reacts with molecular oxygen (O_2_) with the formation of superoxide (O_2_^−•^) and subsequent ROS overproduction in the mitochondrial matrix. Similar mechanism is reported for Complex III. PQ-induced alterations in electron transport chain ultimately results in reduction in mitochondrial transmembrane potential and reduced ATP synthesis by Complex V. Overaccumulation of mitoROS mediates PQ-induced lipid peroxidation, mtDNA damage, mPTP opening, and cytochrome c leakage with subsequent induction of apoptosis.

Paraquat increased the number of fragmented mitochondria and reduced the membrane potential, the activity of Complexes I–IV, and the levels of pyruvate and lactate, whereas ATP production was not affected. In addition, after paraquat treatment, the transcript levels of marker genes were significantly upregulated, which implies a close connection between mitochondrial dysfunction and the oxidative stress response [192]. Finally, recent reports demonstrate that chronic multigenerational exposure of *C. elegans* to mitochondrial toxicants such as paraquat can affect reproduction and aging by a mitochondrial dysfunction-altering dynamic, probably through elevated ROS production that leads to oxidative damage to the DNA [193]. The effects of paraquat on mitochondrial functions are demonstrated in Figure 2.

#### 3.3.2. Rotenone

Rotenone is a highly toxic, naturally occurring botanical pesticide mainly used in organic farming. Due to its ability to inhibit mitochondrial Complex I and to create an oxidative stress environment in the cell, rotenone is used as a chemical stressor in the study of PD and to recapitulate hallmarks of parkinsonism and has selectivity for dopaminergic neurons [194,195,196]. The *C. elegans* response to rotenone involves multiple metabolic changes that might result in compensation without significant changes in oxygen consumption or steady-state ATP levels [17,197]. Transgenic α-synuclein worms decreased the cell viability and reduced mitochondrial respiration in response to rotenone, with no change in the structural features of the dopaminergic neurons’ mitochondria [198]. Additionally, mitochondrial DNA replication is suppressed by rotenone, pointing out the role of mtDNA biogenesis and mitochondrial content in the process of dopaminergic neuron damage and degeneration in *C. elegans* [199].

#### 3.3.3. Thiocarbamates (and Benomyl)

The thiocarbamate pesticides have been used since 1940 and are considered of low or intermediate toxicity, although they contain chelated metals including Mn (maneb), Mn/Zn (mancozeb), and other elements that can be released as potentially toxic ions or even other toxic metabolites as isothiocyanates and ethylene-thiourea [200].

Benomyl is a benzimidazole fungicide widely used in agriculture that, although restricted in the USA and Europe, is still used in developing countries. It undergoes bioactivation to S-methyl N-butyl thiocarbamate sulfoxide, a potent aldehyde dehydrogenase (ALDH) inhibitor in liver and brain mitochondria [201,202,203], thus inducing oxidative stress and apoptosis in neural cells [204]. There is evidence that Mn/Zn ethylene-bis-dithiocarbamate derivatives impair locomotion, and induce damage to both GABAergic and dopaminergic neurons in the model organism *C. elegans* [205,206,207]. In addition, this compound is responsible for mitochondrial Complex I inhibition and concomitant H_2_O_2_ production [208,209].

Similar to disulfiram [210], environmental exposure to thiocarbamates promotes cellular damage by direct inhibition of the ALDH isoenzymes associated with the accumulation of toxic aldehydes, particularly DOPAL (3,4-dihydroxyphenylacetaldehyde) and 4-HNE (4-hydroxy-2-nonenal) [211,212], evidencing the crucial role of these isoenzymes in several pathologies [213]. Interestingly, ALDH inhibition results from the 4-HNE-cysteine adduct formation in the active site of ALDH, a process reversible at low 4-HNE concentrations and irreversible at higher ones [214]. DOPAL is toxic via several mechanisms: protein cross-linking oxidation to quinones, hydroxyl radical production, and increased toxicity exerted by other agents [215]. Thus, the NAD(P)-dependent ALDH superfamily is considered a critical step in the removal of toxic aldehydes and its dysfunction has been associated with pesticide exposure, aging, and neurodegenerative diseases, particularly PD [216,217,218,219]. Both mitochondrial ALDH2 and cytosolic ALDH1A1 catalyze DOPAL to a 3,4-dihydroxyphenylacetic acid (DOPAC) formation, which in most tissues, except substantia nigra, is ultimately converted by the enzyme catechol o-methyltransferase (COMT) to homovanillic acid (HVA), the end product of the dopamine metabolism [217,220,221].

#### 3.3.4. Organophosphates and Carbamates

Organophosphate compounds, the oldest chemicals used as warfare nerve agents in the 1940s, are widely used in insect control [222]. The primary effect of these compounds is the inhibition of the acetylcholinesterase enzyme, leading to acetylcholine buildup and a subsequent failure of transmission, but other mechanisms such as the disruption of energetics and redox signaling have also been documented [223,224]. Importantly, the mitochondrial effects of some organophosphates require enzymatic activation to the oxon form. In *C. elegans*, Williams and Dusenbery (1990) were the first to report a reduction in movement in nematodes exposed to malathion and dichlorvos at high, but not environmentally relevant exposure levels [225]. While feeding behavior, brood size, and body length were also affected, it was only recently that redox status and mitochondrial respiration attracted attention as endpoints in pesticide exposure in *C. elegans* [226]. In effect, Leung et al., 2019, demonstrated mtDNA damage after exposure to chlorpyrifos in *C. elegans*, although is not clear whether this occurs at levels below acetylcholinesterase inhibition [227]. In another report, phoxim (O, O-diethyl O-(alpha-cyanobenzylideneamino), phosphorothioate) an organic phosphorus pesticide, and carbaryl (1-naphthyl methylcarbamate), a carbamate insecticide, caused oxidative stress and altered the antioxidant enzyme activities and their gene expressions in *C. elegans* [228]. Finally, quinalphos (O, O-diethyl O-quinoxalin-2-yl phosphorothioate), a synthetic organophosphorus pesticide, altered oxidative stress-related genes which the authors propose as biomarkers for monitoring quinalphos exposure.

#### 3.3.5. Pyrethrins and Pyrethroids

Pyrethroids and their synthetic derivatives, pyrethrins, produce prolonged depolarization of the nerve membrane acting on the opening of the sodium channels of the nerve. Among the relatively few studies described in *C. elegans*, deltamethrin reduced survival in a concentration-dependent manner affecting the expression of voltage-gated calcium channel α1 subunits, locomotion, egg-laying, and foraging behavior [229]. Cypermethrin, a-cyano-3-phenoxybenzyl ester of 2,2-dimethyl-3-(2,2-dichloro vinyl)-2-2-dimethyl cyclopropane carboxylate induces oxidative stress by increasing free radicals, decreasing reduced glutathione levels, increasing protein carbonyl levels and altering the activities of antioxidant enzymes [230], thus altering the redox status; an effect that merits further attention in terms of the mitochondrial participation.

#### 3.3.6. Glyphosate

Glyphosate (N-phosphonomethyl-glycine) is the active ingredient of a herbicide extensively used in the world. The mechanism of action is related to the inhibition of 5-enolpyruvylshikimate-3-phosphate synthase, an enzyme involved in the synthesis of aromatic amino acids in plants [231]. Although glyphosate itself is relatively non-toxic, its commercially available formulations affect survival, locomotion, and fertility, and induce changes in the gene expression of the antioxidant enzymes in *C. elegans* [232]. Other reports demonstrated that glyphosate formulations induced neurodegeneration in both dopaminergic and GABAergic neurons that along with redox imbalance and mitochondrial dysfunction are a hallmark of neurological diseases [206,207]. Interestingly, chronic glyphosate formulations administered to *C. elegans* increased H_2_O_2_ production and glutathione sulfur transferase-4 (GST-4) upregulation along with mitochondrial inhibition as evidenced by reduced oxygen consumption, proton gradient, and ATP production, probably due to Complex II inhibition [233,234]. Importantly, ROS production, the clt-1 gene, and catalase activity are considered excellent biomarkers to assess the environmental risk of glyphosate use in glyphosate formulation-treated nematodes [235].

#### 3.3.7. Triazines (Atrazine)

Although now banned in the European Union, atrazine is still the most applied herbicide in the world [236]. Reports on *C. elegans* indicated that relatively low atrazine concentrations increased the ROS levels and decreased locomotion behavior. Exposure to higher concentrations reduced body length, life expectancy, and brood size. In addition to these alterations, atrazine activates the mitochondrial unfolded protein response, as well as increases the mitochondrial damage and vacuolar degeneration associated with a decrease in mitochondrial cristae and volume density [237].

#### 3.3.8. Organochlorines (Lindane)

Lindane (g-hexachlorocyclohexane), is an organochlorine that was widely used between 1950 and 1980 in medical and agricultural products [238]. Chronic exposure to lindane significantly influenced the expression of genes related to oxidative stress and cell apoptosis (*isp-1*, *sod-3*, *ced-3*, and *cep-1* genes), indicating that oxidative stress and cell apoptosis could play an important role in the toxicity induced by this pesticide in nematodes [239].

#### 3.3.9. Neonicotinoids

These conform to a class of insecticides structurally similar to nicotine and are the most widely used insecticides today with reported low toxicity in other organisms. Using *C. elegans* as an animal model, it has been shown that exposure to neonicotinoid insecticides could result in oxidative stress and damage to reproduction, locomotion behaviors, and growth [240]. Later reports ascribed reduced growth in nematodes, lower fecundity as measured by increased germline apoptosis, a decrease in egg-laying, and fewer viable offspring to neonicotinoid formulations [241].

#### 3.3.10. Other Pesticides

Dithianon is a fungicide with thiol-reactivity, that causes concentration-dependent neurotoxicity of dopaminergic neurons and neurobehavioral impairments in *C. elegans*, as well as increases in oxidative stress and mitochondrial fragmentation, which are strongly linked to PD pathology [242]. Fluopyram (N-{2-[3-chloro-5-(trifluoromethyl)-2-pyridyl]ethy-α,α,α-trifluoro-o-toluamide) is a fungicide member of the pyramid group that at sublethal rates induced oxidative stress through an increase in ROS production and a decrease in antioxidant enzyme activities and glutathione (GSH) content, leading to an oxidative imbalance in *C. elegans* [243].

## 4. Conclusions

Mitochondria and their role in cellular function have unveiled their significance as the powerhouses of cells. Their involvement in cellular respiration and the regulation of various processes highlights their crucial role in maintaining cellular homeostasis. In this context, *C. elegans* has emerged as an important model organism in the field of mitochondrial research. Its genetic similarity to mammals, coupled with its simple and well-characterized biology, has made it a preferred choice for studying the effects of contaminants and drugs on mitochondrial function. By utilizing *C. elegans*, studies have made significant improvements in elucidating the underlying mechanisms of the mitochondrial dysfunctions observed in neurodegenerative disorders and behavioral alterations. *C. elegans* as an experimental model offers several advantages, such as short lifespan, small size, and ease of cultivation, all of which allow for rapid experimentation and large-scale screening, facilitating the identification of potential therapeutic targets and toxicological effects. Additionally, the transparency of *C. elegans* embryos enables real-time observation of the mitochondrial dynamics and responses to various stressors, providing valuable insights into the intricate effects of contaminants and drugs on mitochondrial function. Table 1 summarizes the potential mechanism by which each xenobiotic impairs mitochondrial function.

The findings obtained from studying mitochondrial function in *C. elegans* has not only enhanced our understanding of the underlying molecular mechanisms, but they may also contribute to the development of novel therapeutic strategies to mitigate mitochondrial dysfunction-related diseases. Furthermore, the use of *C. elegans* as a model system provides a bridge between basic research and translational medicine, allowing for the rapid translation of experimental findings into potential clinical applications. It represents a powerful platform for investigating the complex interplay between heavy metals, drugs, pesticides and mitochondrial processes, ultimately contributing to the development of effective interventions to combat mitochondrial-related disorders and improve human health. Further, future studies should explore the full spectrum of interactions between various stressors and mitochondrial processes, leading to a better understanding of how these factors impact cellular health. Additionally, this research might aid in developing innovative strategies to counteract the adverse effects of these stressors on mitochondria.

## Figures and Tables

**Table 1:** Potential mechanism by which each xenobiotic impairs mitochondrial function.

Xenobiotic	Potential Mechanism of Impairment of Mitochondrial Function	Reference
As	Low-dose arsenite increases ROS formation, promoting upregulation of mitochondrial proteins, increasing the lifespan while higher concentrations reduce longevity.	[86]
As	Reduced ATP-like respiration, spare respiratory capacity and augmented proton leak	[87]
As	Disrupted mitochondrial function in fusion-deficient worms, suggesting that disruption of pyruvate metabolism and Krebs cycle activity trigger the mitochondrial deficits	[66]
Cd	*pink-1* strain showed higher levels of mtDNA damage	[103]
Cd	Reduced OCR in a dose-dependent manner; Cd exposure is positively associated with worm growth inhibition	[108]
Cd	Transcriptional alterations in several genes related to ATP turnover and mitochondrial biogenesis and functioning	[109]
Mn	Dysfunctions of mitochondrial genes (e.g., *PINK1*)	[116]
Hg	Loss of dopaminergic neurons, observed in Parkinson’s later in life, following early-life (L1) exposure	[122]
Fe	Mitoferrin-1 led to a decrease in mitochondrial Fe content and a reduction in mitochondrial ROS	[126]
Fe	Impaired mitochondrial energy production and protein balance	[15]
Ethanol	Fragmented mitochondria, probably because of fission from the internal membrane	[169]
Ethanol	*Dauer* larvae survive much longer because during energy depletion EtOH prevents or delays mitochondrial fragmentation and deterioration	[170]
Paraquat	Structural damage in mitochondria, ATP depletion, and increased autophagy	[191]
Paraquat	Increased number of fragmented mitochondria and reduced membrane potential, Complexes I–IV activity, and pyruvate and lactate levels	[192]
Paraquat	Elevated ROS production that leads to oxidative damage to the DNA	[193]
Rotenone	Loss of Complex I function including upregulation of mitochondrial Complexes II and V, activation of the glyoxylate pathway, glycolysis, and fatty acid oxidation	[197]
Rotenone	Mitochondrial DNA replication is suppressed, pointing out the role of mtDNA biogenesis and mitochondrial content in the process of dopaminergic neuron damage	[198]
Thiocarbamates	Mitochondrial Complex I inhibition and increased ROS	[208]
Thiocarbamates	Mitochondrial dysfunction and increased ROS production	[209]
chlorpyrifos	mtDNA damage after exposure to chlorpyrifos	[227]
Organophosphates and carbamates	Oxidative stress altered the antioxidant enzyme activities and their gene expressions	[228]
Pyrethrins and Pyrethroids	Oxidative stress by increasing free radicals, decreasing GSH levels, increasing protein carbonyl levels and altering the activities of antioxidant enzymes	[230]
Glyphosate	Reduced oxygen consumption, proton gradient, and ATP production	[233]
Glyphosate	Inhibition of Complex II and increased hydrogen peroxide levels	[234]
Triazines (atrazine)	Mitochondrial unfolded protein response, increased mitochondrial damage and vacuolar degeneration, associated with a decrease in mitochondrial cristae and volume density	[237]
Organochlorines (lindane)	Expression of genes related to oxidative stress and cell apoptosis (*isp-1*, *sod-3*, *ced-3*, and *cep-1* genes)	[239]
Dithianon	Increases in oxidative stress and mitochondrial fragmentation	[242]
Fluopyram	Increase in ROS production and decrease in antioxidant enzymes activities, and GSH content	[243]

## Data Availability

Not applicable.
